# In conditions of over-expression, WblI, a WhiB-like transcriptional regulator, has a positive impact on the weak antibiotic production of *Streptomyces lividans* TK24

**DOI:** 10.1371/journal.pone.0174781

**Published:** 2017-03-30

**Authors:** Lan Yan, Qizhong Zhang, Marie-Joelle Virolle, Delin Xu

**Affiliations:** 1 Department of Ecology, Institute of Hydrobiology, School of Life Science and Technology, Key Laboratory of Eutrophication and Red Tide Prevention of Guangdong Higher Education Institutes, Engineering Research Center of Tropical and Subtropical Aquatic Ecological Engineering, Ministry of Education, Jinan University, Guangzhou, PR China; 2 Group "Energetic Metabolism of *Streptomyces* ", Institute for Integrative Biology of the Cell (I2BC), CEA, CNRS, Univ. Paris‐Sud, INRA, Université Paris‐Saclay, Gif‐sur‐Yvette Cedex, France; Beijing Institute of Microbiology and Epidemiology, CHINA

## Abstract

Regulators of the WhiB-like (*wbl*) family are playing important role in the complex regulation of metabolic and morphological differentiation in *Streptomyces*. In this study, we investigated the role of *wblI*, a member of this family, in the regulation of secondary metabolite production in *Streptomyces lividans*. The over-expression of *wblI* was correlated with an enhanced biosynthesis of undecylprodigiosin and actinorhodin and with a reduction of the biosynthesis of yCPK and of the grey spore pigment encoded by the *whiE* locus. Five regulatory targets of WblI were identified using *in vitro* formaldehyde crosslinking and confirmed by EMSA and qRT-PCR. These included the promoter regions of *wblI* itself, two genes of the ACT cluster (*actVA3* and the intergenic region between the divergently orientated genes *actII-1* and *actII-2*) and that of *wblA*, another member of the Wbl family. Quantitative RT-PCR analysis indicated that the expression of *actVA3* encoding a protein of unknown function as well as that of *actII-1*, a TetR regulator repressing the expression of *actII-2*, encoding the ACT transporter, were down regulated in the WblI over-expressing strain. Consistently the expression of the transporter *actII-2* was up-regulated. The expression of WblA, that is known to have a negative impact on ACT biosynthesis, was strongly down regulated in the WblI over-expressing strain. These data are consistent with the positive impact that WblI over-expression has on ACT biosynthesis. The latter might result from direct activation of ACT biosynthesis and export and from repression of the expression of WblA, a likely indirect, repressor of ACT biosynthesis.

## Introduction

*Streptomyces* are Gram-positive, filamentous soil bacteria of considerable biotechnological importance. Indeed this genus produces two thirds of all known antibiotics as well as other bio-active molecules, including antitumor agents, immune-suppressants, apoptosis inducers and antifungals, herbicides, insecticides etc… used in medicine or agriculture [1]. These bacteria are characterized by a complex developmental cycle that starts, when the nutritional conditions are favorable, by the germination of spores that develop into a substrate mycelium. Subsequently, some still poorly defined signals of nutrient limitation, are thought to trigger the development of aerial hyphae from the substrate mycelium. The tip ends of the aerial hyphae differentiate into uni-genomic spores and the production of a grey pigment encoded by the *whiE* locus accompanied the complete differentiation process [2,3]. This complex morphological development is mainly under the control of the *bld* and *whi* genes, that are required for the formation of aerial mycelium and spores, respectively [2,3].

The complex *bld* signaling cascade has been extensively studied leading to the characterization of the BldA, BldD, BldH and BldN regulons [4–8]. A signal molecule c-di-GMP was recently shown to induce the dimerization of the regulator BldD that is necessary to activate its DNA-binding activity [9,10]. The binding of BldD results in the repression of the sporulation genes during vegetative growth leading to a delay in the differentiation process [9,10]. One of the genes under the negative control of BldD is the σ factor, *whiG* [4,11,12]. The latter is necessary for the expression of numerous genes of the *whi* cascade, including that of the regulators *whiA*, *whiH*, *whiI* but not *whiB* [12–15]. Whi regulators are named from the white color of aerial hyphae following the mutation/disruption of their cognate encoding genes. They are known to play key roles in the differentiation of aerial hyphae into mature spores [2]. WhiB, regarded as the founding member of the WhiB-like (Wbl) family proteins, is one of the Whi regulators that is necessary for the septation steps preceding sporulation [16,17]. A recent genome-wide study demonstrated that WhiB is a transcriptional factor that binds, in cooperation with WhiA, upstream of nearly 240 transcription units required for developmental cell division [18]. Wbl proteins usually contain an unconventional helix-turn-helix motif and a [4Fe-4S] iron-sulfur cluster that have the ability to detect redox changes and regulate gene expression accordingly [19]. Such redox sensing clusters have been shown to play diverse and critical roles in actinobacterial biology, including morphological differentiation, antibiotic production, antibiotic resistance and pathogenesis [16,20–22]. There are 14 WhiB-like (Wbl) regulators present in *S*. *coelicolor*, 11 Wbl proteins are encoded by chromosomal genes, and 3 are encoded by the plasmid SCP1 [23]. *wblA* is the most extensively studied of these regulators. It was shown to play important role in the early stage of aerial hyphal development [23,24] and to have a negative impact on oxidative stress response [25,26] and antibiotic production [24]. In contrast WhiD was shown to be essential for pre-spore maturation [27–30].

In this study we characterized another member of the Wbl gene family, WblI (SCO5046). This gene was previously shown to be a target of SCO3201, a regulator of the TetR family, whose overexpression led to strong repression of both antibiotic production and sporulation in *S*. *coelicolor* [31]. We demonstrated that the over-expression of the homologue of WblI in *S*. *lividans* TK24, greatly enhanced the weak ability of this strain to synthetize undecylprodigiosin (RED) and actinorhodin (ACT), peptidyl and polyketide secondary metabolites, respectively whereas it had a negative impact on the biosynthesis of yCPK, a type I polyketide as well as of the grey spore polyketide pigment encoded by the *whiE* locus. Regulatory targets of WblI were identified by formaldehyde cross linking and confirmed by EMSA and qRT-PCR. WblI constitutes a new player in the complex regulatory network governing secondary metabolite production and morphological differentiation in *Streptomyces*. A regulatory model consistent with the impact that WblI over-expression/deletion has on the expression of its targets and on antibiotic production is proposed and discussed. This model clarifies the hierarchical relationships between WblI and WblA.

## Materials and methods

### Bacterial strains, media and culture conditions

*S*. *lividans* TK24 (*str-6*, SLP2-, SLP3-) was used in this study. *Escherichia coli* DH5α and BL21 were used as the hosts for routine subcloning and protein expression, respectively. SFM [32] and GYM [33] media were used for spore collection and the assessment of spore grey pigment production, respectively. R2YE medium, traditionally used for protoplast regeneration, was used for solid-grown cultures of *S*. *lividan*s. To assess antibiotic production, 10^7^ spores of various *Streptomyces* strains were spread on top of cellophane discs on R2YE agar medium. When necessary, ampicillin, kanamycin or apramycin were added to the culture medium at 50 μg/ml, whereas thiostrepton was added to R2YE liquid medium at 5 μg/ml. Unless otherwise stated, *E*. *coli* and *Streptomyces* strains were incubated at 37°C and 28°C, respectively, and a shaking speed of 220 rpm was maintained for liquid culture.

### Construction of *S*. *lividans* TK24 strains overexpressing or disrupted for *wblI*

In order to overexpress *wblI* in *S*. *lividans* TK24, the *wblI* gene was amplified from the genomic DNA of *S*. *lividans* TK24 by PCR using primers ExpWblI-BamHI and ExpWblI-HindIII. The PCR products were digested by *Bam*HI and *Hind*III, and cloned into pWMH3-ermE*, a derivative of high-copy-number vector pWHM3. This contains the strong, constitutive ermE* promoter, an efficient ribosomal binding site and confers resistance to thiostrepton [34]. The constructed pWHM3-ermE*-wblI and the control empty vector pWHM3-ermE* were transformed into *S*. *lividans* TK24 protoplasts and transfornants were selected in the presence of thiostrepton at 50 μg/ml. A method of in-frame deletion [32] was used to construct the *wblI* deletion mutant. Two 1.5 kb DNA fragments flanking the *wblI* coding region were amplified from the *S*. *lividans* TK24 genomic DNA by PCR using primer pairs WblI-Up1/WblI-Up2, WblI-Down1/WblI-Down2. The resulting two PCR fragments were individually digested with the corresponding restriction enzymes, and subsequently cloned into pDH5 [32] cut by *Hind*III and *Eco*RI using a triple ligation strategy, giving pDH5-Δ*wblI*. The resulting plasmid was used to delete *wblI* using the following procedure: protoplasts of *S*. *lividans* TK24 transformed with pDH5-Δ*wblI* were regenerated on R2YE medium without antibiotic selection at 28°C until fully sporulating. Spores were harvested and plated on R2YE medium in order to have well isolated colonies. Once sporulated these well-separated colonies were replica plated on R2YE and R2YE containing thiostrepton (50 μg/ml) to identify the thiostrepton-sensitive colonies. The chromosomal structure of the wild-type strain and the thiostrepton-sensitive colonies was compared in the *wblI* region by PCR, using primers ExpWblI-NdeI and ExpWblI-XhoI. The near-complete deletion of *wblI* gene in *S*. *lividans* TK24 was verified by the size of the amplified PCR products (396 bp for wild type strain, 96 bp for mutant strain).

### Quantification of RED and ACT production

Quantification of undecylprodigiosin (RED) and actinorhodin (ACT) (both intra and extracellular) production was carried out as documented previously [31]. Briefly, 10^7^ spores of various *Streptomyces* strains were spread on the surface of cellophane discs laid on R2YE plates and cultivated at 28°C for 72 h. In order to assay the cellular bound RED and intracellular ACT, approximately 50 mg of mycelium (dry weight) was collected and extracted by vortexing for 30 min at 4°C in 1 ml methanol and in 1 ml KOH (1N) respectively. For RED assay, the extract was acidified to pH 2~3 by HCl and the OD_530nm_ was determined using a spectrometer (SHIMADZU). To assay intracellular ACT, the OD_640nm_ of the extract was measured using the same spectrometer. For extracellular ACT determination, one-quarter of an 8 cm diameter plate of the agar R2YE medium was collected, smashed and immersed in 10 ml H_2_O for 24 h at 4°C. The mixture was centrifuged and the resulting supernatant was transferred into a fresh tube and 10 ml KOH (1M) was added. After gentle inversion, 10 ml of HCl (3M) was added and the mixture incubated on ice for 10 min then centrifuged. The supernatant was discarded and the pellet containing ACT was re-suspended in 1 ml of KOH (1M) and the OD_640nm_ of the resulting solution was determined. Each sample was processed in triplicate.

### Overexpression and purification of Histidine-tagged WblI in *E*. *coli*

The *wblI* coding sequence was amplified from the genomic DNA of *S*. *lividans* TK24 by PCR using primers ExpWblI-NdeI and ExpWblI-XhoI. The resulting DNA fragments were cloned into pET22b(+) cut by *Nde*I and *Xho*I, and the resultant plasmid transformed into *E*. *coli* BL21, a suitable host for protein expression. The resulting transformants were cultivated in LB medium supplemented with 50 μg/ml ampicillin at 37°C until OD_600_ reached 0.6. IPTG was then added at a final concentration of 1 mM, and incubation was pursued for 6 extra hours. The cells were collected by centrifugation at 12,000 g for 10 min. Then the cell pellet was resuspended in lysis buffer (50 mM Na_2_HPO_4_, pH 8.0, 500 mM NaCl) and sonicated (Hielscher Ultrasonics UP400S, 0.5 cycle and 20% amplitude) on ice until reaching complete homogeneity. After centrifugation at 14,000 rpm for 20 min, the cell extract was saved and passed through an Ni-NTA column (Cat. No. 30210; Qiagen) on a Biologic LP apparatus (Bio-Rad). The six-histidine-tagged WblI (His_6_-WblI) was purified to near homogeneity according to the manufacturer’s instructions.

### Isolation of the putative WblI interacting targets

The putative WblI binding targets were isolated as described previously [35]. Briefly, 100~200 pmol of purified His_6_-WblI was incubated with 10 pmol of *S*. *lividans* TK24 genomic DNA for 15 min at room temperature in the binding buffer, total volume of 1 ml. A negative control reaction that contained only genomic DNA, free of WblI, was carried out in parallel. The binding reaction was fixed by addition of 1 ml of crosslinking buffer (HEPES, NaCl, EDTA, 37% formaldehyde) and incubated at 37°C for 10 min then at 4°C for 1 h. Genomic DNA was sheared to an average size of 2~3 kb by sonication for 8 sec on ice (Hielscher Ultrasonics UP400S, 0.5 cycle and 20% amplitude). The complex of His_6_-WblI crosslinked with fragments of genomic DNA was isolated by passing through an Ni-NTA Agarose (Cat. No. 30210; Qiagen) column. The de-crosslinking of WblI-DNA was carried out by incubation overnight with 200 mM NaCl at 65°C for 4 h and proteinase K (at a final concentration of 20 μg/ml). The de-crosslinked DNA was recovered by EtOH precipitation. The resulting DNA pool was digested by *Sau3*AI, and subsequently cloned into pUC18 cut by *Bam*HI. The inserted DNA fragments were then identified by nucleotide sequencing. The promoters present on the sequenced fragments and/or its flanking regions (within 2~3 kb) were retained for further analysis.

### Electrophoretic mobility shift assay (EMSA)

The promoter regions of *wblA*, *wblI*, *actVA3* and the intergenic region between *actII-1* and *actII-2* were amplified from *S*. *lividans* TK24 genomic DNA by PCR using primer pairs BS3579F/BS3579R, BS5046F/P5046R, BS5078F/BS5078R, BS82-83F/BS82-83R (Table 1), respectively. The resulting PCR products were 5’ labeled with FITC by PCR using primer Plabel (Table 1). 50 pmol of each of the promoter regions was incubated with purified His_6_-WblI in various concentrations for 15 min at room temperature in binding buffer (10 mM Tris-HCl, 50 mM KCl, 1mM DTT, pH 7.5), total volume of 20 μl. For the competition assay, excess amounts of specific competitors of unlabeled *wblI* promoter or non-specific competitors of an unlabeled unrelated DNA probe were introduced. After incubation, the reaction mixtures were resolved on a 5% native polyacrylamide gel pre-run at 100 V for 30 min and run at 100 V for 90 min in a running buffer containing 45 mM Tris-HCl, pH 8.3, 45 mM boric acid, 10 mM EDTA. Visualization of the DNA signal was carried out by fluorescence imaging using a UVI Alliance 4.7 imager (UK).

**Table 1:** Synthetic oligonucleotides used in this study.

Primer	5’ → 3’ sequence*^a^*	Positions*^b^*	Purpose
**ExpWblI-BamHI**	**ATAAAAGGATCCTGACGCATCGTCTCTCGCTAGC**	**-46 to +406**	**Amplification of the *wblI* coding sequence for overexpression**
**ExpWblI-HindIII**	**ATAAAAAAGCTTAGAGGGTGCCCTTTCGGGTG**		
**WblI-Up1**	**ATAAAAAAGCTTTTCTCCTCCCACATGGTCAG**	**-1500 to +80**	**Amplification of the 1.5 kb fragment located upstream of *wblI***
**WblI-Up2**	**ATAAAATCTAGATCTTGGTCCCTGTCCCGC**		
**WblI-Down1**	**ATAAAATCTAGAGAACTCAGGAACGCCGCC**	**+327 to +1860**	**Amplification of the 1.5 kb fragment located downstream of *wblI***
**WblI-Down2**	**ATAAAAGAATTCAGGACGATGACCCCGAGG**		
**ExpWblI-NdeI**	**ATAAAACATATGGTGCTGCAACCGCCGCATTCGTC**	**+1 to +23** **+357 to +375**	**Amplification of *wblI* coding sequence for protein expression**
**ExpWblI-XhoI**	**ATAAAACTCGAGGCCGGCCGCCGCTATGCG**		
**BS3579F**	**AGCCAGTGGCGATAAGCGTATCAATACGTCCGGCGA**	**-377 to +51**	**Amplification of the *wblA* promoter for EMSA**
**BS3579R**	**AGCCAGTGGCGATAAGATCGGTAGTGCGGCAGGC**		
**BS5046F**	**AGCCAGTGGCGATAAGCGAGTACCAGCAGGTCGTCA**	**-385 to +20**	**Amplification of the *wblI* promoter for EMSA**
**BS5046R**	**AGCCAGTGGCGATAAGCTACCTGCAGGGACGAAT**		
**BS5078F**	**AGCCAGTGGCGATAAGGTCTCGTTCCGCGTCAACAC**	**-395 to +8**	**Amplification of the *actVA3* promoter for EMSA**
**BS5078R**	**AGCCAGTGGCGATAAGGTACTCATCCAGCCGCCCT**		
**BS82-83F**	**AGCCAGTGGCGATAAGCGACACGTGCTCCTCATCGT**	**-116 to +89 (relative to the ACTII-2 translation start as +1)**	**Amplification of the *actII-1/actII-2* promoter for EMSA**
**BS82-83R**	**AGCCAGTGGCGATAAGGTTCCGTCCGGTCCGGGG**		
**Plabel**	**AGCCAGTGGCGATAAG**	****	**FITC labeling**
**RT3579F**	**ATGGGCTGGGTAACCGACTG**	**+1 to +80**	**RT-PCR of *wblA***
**RT3579R**	**GCTGCTCCCTGAACGAACAG**		
**RT5078F**	**CCATCCGTACGACGACGTGA**	**+357 to +474**	**RT-PCR of *actVA3***
**RT5078R**	**CCGGCTGTGTGTGAGGAAGA**		
**RT5082F**	**CAGGAGCTGAAGACCGGACA**	**+175 to +320**	**RT-PCR of *actII-1***
**RT5082R**	**ATCTCCTTGACCTGCTCCGC**		
**RT5083F**	**ATGACGCCCTGGTCGGTGTT**	**+1027 to +1173**	**RT-PCR of *actII-2***
**RT5083R**	**CTGGTCGCCGATCGTCAACAGCAT**		
**RT-hrdB-F**	**AGGACGGCGACAGCGAGTTC**	**+1235 to +1394**	**RT-PCR of *hrdB***
**RT-hrdB-R**	**CCGAAGCGCATGGAGACG**		

^***a***^ Underlined nucleotides show no homology to the template; they were used for FITC labeling.

^*b*^ Positions relative to the translational start site as +1.

### Isolation of total RNA and RT-PCR

*Streptomyces* mycelia were collected from three independent R2YE plates at various time points and total RNA isolated using an RNAprep kit (TIANGEN, Cat. No. DP430) according to the manufacturer’s instructions. Two micrograms of each RNA sample were used as a template for the first strand cDNA synthesis for various genes using gene specific primers of RT3579R, RT5078R, RT5082R, RT5083R and RT-hrdB-R, respectively. Quantitative real-time PCR was performed in a 20 μl reaction mixture comprising SYBR Green Real-time PCR Master Mix (Toyobo, Japan), ten percent of the cDNA synthesis reaction mixture (2 μl), each of the gene-specific primer pairs, including RT3579F/RT3579R, RT5078F/RT5078R, RT5082F/RT5082R, RT5083F/RT5083R and RT-hrdB-F/RT-hrdB-R as internal control. Real-time PCR was run on an iCycler iQ instrument (Bio-Rad, USA.) following the manufacturer’s instructions. The PCR cycling conditions were 95°C for 4 min, 35 cycles at 94°C for 30s, 62°C for 30s, 72°C for 20s and a gradient from 65°C to 95°C for 10 min under a continuous monitoring. *hrdB*, an housekeeping gene encoding the major vegetative sigma factor whose expression is constant throughout growth, was used as an internal control to normalize the relative expression of each target gene.

## Results

### WblI over-expression has a positive impact on RED and ACT biosynthesis

**Fig 1:**
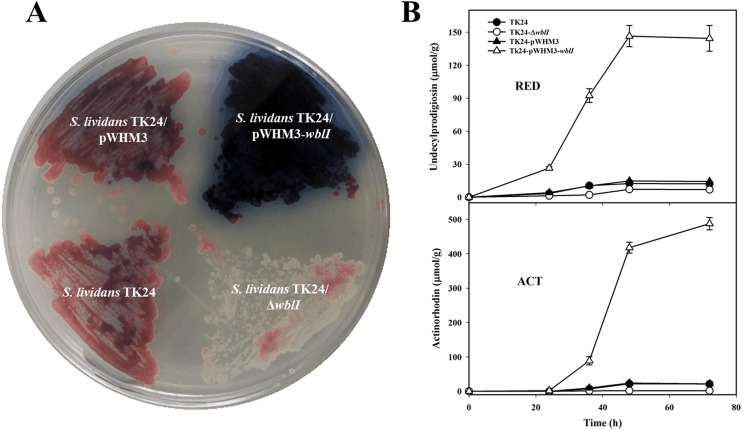
Impact on secondary metabolite production by the introduction of the plasmids pWHM3, pWHM3-*wblI* and of the deletion of *wblI* (Δ*wblI*) in *S*. *lividans* TK24. (A) Picture of patches grown on R2YE medium for 72 h; (B) Quantitative analysis of RED and ACT productions, all values were expressed as means ± SD (n = 5).

The production of RED and ACT was assayed in the original strain, *S*. *lividans* TK24, in the strain deleted for *wblI* and in derivatives of *S*. *lividans* TK24 carrying the empty plasmid pWHM3-ermE* or the plasmid pWHM3-ermE*-*wblI*. Results are shown in Fig 1. The over-expression of WblI, led to strong enhancement of the production of RED and ACT, the red hybrid peptide-polyketide and blue polyketide antibiotics usually weakly produced by this strain (Fig 1A) but had no impact on growth (Figure A in S1 Appendix). Quantitative analysis showed that at 72 h, *S*. *lividans* TK24 over-expressing *wblI* produces 10 and 23 fold more RED and ACT, respectively than the control strain (Fig 1B). Similar results were obtained when WblI of *S*. *coelicolor* (WblI^SC^) was over-expressed (data not shown). WblI and WblI^SC^ differ by one amino acid located outside of the WhiB helix turn helix domain (Figure B in S1 Appendix). As anticipated, the deletion of *wblI* had little (or a slight negative) impact on the already weak production of these antibiotics in *S*. *lividans* TK24 (Fig 1). These results demonstrated the positive regulatory effect of WblI on ACT and RED biosynthesis in *S*. *lividans* TK24. In order to determine whether this effect was direct or indirect, attempts were made to isolate putative regulatory targets of WblI using *in vitro* formaldehyde crosslinking.

**Fig 2:**
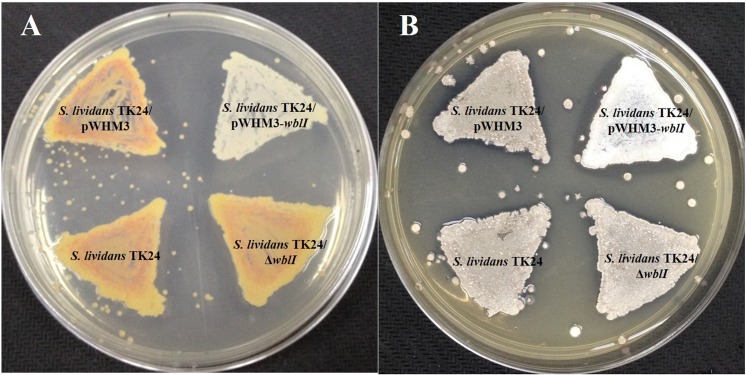
**Effects of *wblI* overexpression and deletion on the production of yCPK (A) and spore grey pigment (B).** Strains were cultivated on GYM medium for 36 h (A) and 72 h (B), respectively.

In contrast, the over-expression of WblI was shown to be correlated with the reduced synthesis of a yellow pigment (Fig 2A) thought to be the metabolic product of the *cpk* gene cluster regulated by the γ-butyrolactone signaling molecule SCB1 [36–38]. We noticed that the spores generated by *S*. *lividans* TK24/pWHM3-ermE*-wblI remained white even upon prolonged incubation and never developed the usual grey color of the control strain *S*. *lividans* TK24/pWHM3 on the GYM solid medium (Fig 2B). However, spores counts with Thomas cell or spores plating in dilution yielded similar number of spores for the two strains (data not shown). This indicated that the over-expression of WblI had little if any impact on morphological differentiation.

### Isolation of the putative regulatory targets of WblI using in vitro formaldehyde crosslinking

*In vitro* formaldehyde cross-linking of the purified His_6_-WblI with its putative targets in the *S*. *lividans* chromosome was performed, followed by the covalent capture of WblI/DNA complex on a Ni-NTA column. After cross-linking reversal, the isolated DNA fragments were cloned into pUC18. Twenty-one clones were obtained and sequenced. Four putative target promoter regions (absent in the negative control, see materials and methods) were identified (Table 2). Two of the targets belong to the ACT biosynthetic gene cluster. These include the region upstream of *actVA3*, a biosynthetic gene of the ACT cluster [39] and the intergenic region between two divergently located genes, *actII-1* and *actII-2*. *actII-1* encodes a TetR regulator known to repress the expression of the divergent gene *actII-2* encoding the ACT transporter [40]. The other targets of WblI were WblI itself and another WhiB-like transcriptional regulator, WblA. The latter was shown to be essential for an early stage of aerial hyphal development [23] and to have a negative impact on the oxidative stress response [25,26] and antibiotic production [24].

**Table 2:** List of putative WblI regulatory targets.

Target gene	Protein	EMSA	In vivo test
*wblA*	WblA, putative transcriptional regulator	√	Down
*wblI*	WblI, putative transcriptional regulator	√	NA
*actVA3*	ActVA3, hypothetical protein of ACT biosynthetic cluster	√	Up
*actII-1*	ActII-1, TetR family transcriptional regulator	√	Down
*actII-2*	ActII-2, probable ACT transporter	√	Up

√: promoter regions shifted up by WblI in EMSA; Up or Down: transcription up or down regulated by overexpressed WblI in RT-PCR; NA: not applicable.

### WblI directly interacts with its regulatory targets in vitro

**Fig 3:**
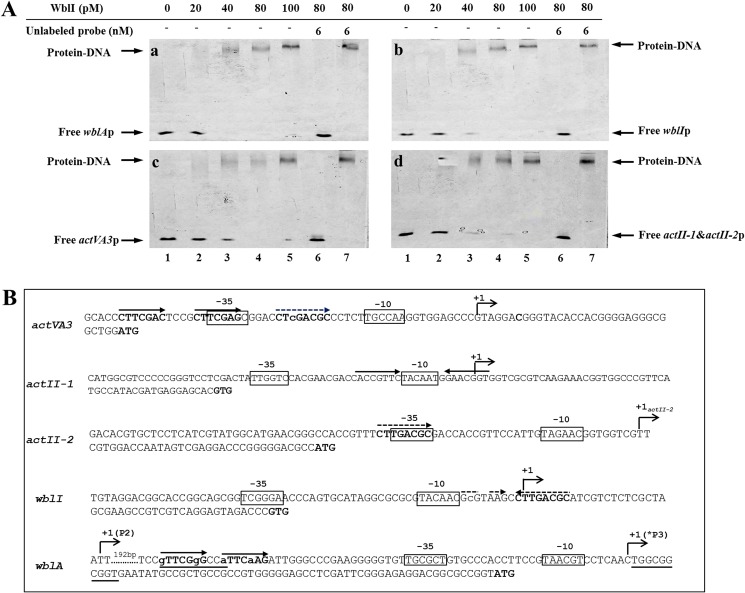
(A) Electrophoretic mobility shift assay of purified WblI with its putative regulatory promoter targets: *wblA* (a), *wblI* (b), *actVA3* (c) and the intergenic region between *actII-1* and *actII-2* (d). In all cases, 50 pmol of FITC-labeled probe was used. The specific (unlabeled target promoters) and non-specific (irrelevant DNA) competitors were introduced in lane 6 and lane 7, respectively. Arrows indicate the positions of DNA-protein complexes and free DNA. (B) Sequence of the promoter regions of *actVA3*, *actII-1*, *actII-2*, *wblI*, *wbl*A. The transcriptional sites are indicated by +1 with bent arrows. Putative -10 and -35 regions are boxed. Translational start codons are in bold. The two related direct or inverted repeats of the sequences CTTCGAS or CTTGACGC thought to constitute WblI operator sites are shown as plain or dotted arrows above the sequence line. AdpA binding motifs are underlined.

In order to confirm the direct interaction of WblI with its four putative targets, regions encompassing the promoter sequences predicted by the online Neural Network Promoter Prediction tool (www.fruitfly.org/seq_tools/promoter.html) were amplified and FITC-labeled by PCR and used as probes for EMSA. Results are shown in Fig 3A. The migration of the promoter fragments of *wblI*, *wblA*, *actVA3*, as well as the intergenic region between *actII-1* and *actII-2* were found to be retarded in the presence of purified WblI in a concentration dependent manner. In all cases, addition of an excess amount of unlabeled probes led to the fading or even disappearance of the shifted bands, whereas the competition assay was largely compromised when an irrelevant DNA probe (+1 to +378 relative to the translational start codon on *wblI* coding sequence) was used. These results demonstrated the functionality of the purified protein as well as the specificity of the protein-DNA interactions.

The four target promoter regions were inspected for the presence of conserved motifs (Fig 3B). Three repeats of closely related sequences CTTCGAS (S standing for G or C) or CTTGACGC were found upstream of the -35 region of *actVA3*. Their position is consistent with the activation of the expression of this gene by WblI. An perfect inverted repeat of the sequence (CACCGTTC TACAATG GAACGGTG) was found bracketing the -10 promoter region of ActII-1(TetR regulator) consistent with the repression of this gene by WblI. In contrast, a single repeat of the sequence (CTTGACGC) that could not constitute an operator site was found overlapping the -35 promoter region of ActII-2 (Act transporter). An imperfect inverted repeat of the sequence CTTGACGC was found downstream of the -10 promoter sequence of *wblI*, suggesting a negative auto-regulation of WblI. Two direct repeats of a degenerated version of the sequence CTTCGAS (gTTCGgG CC aTTCaAG) were found between the P2 and P3 promoters of WblA in overlap with a known negative regulatory site for AdpA [41]. Even though these putative binding sites should be confirmed by foot printing experiments, their localization fits the observed regulatory features of these genes by WblI.

### Assessment of in vivo regulatory effects of WblI using quantitative RT-PCR

**Fig 4:**
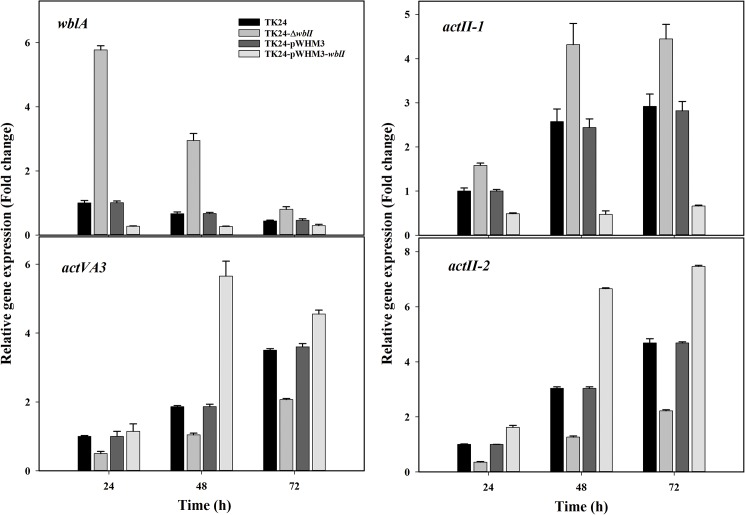
Quantitative analysis of the transcriptional levels of WblI regulatory targets by RT-PCR in the wild type strain of *S*. *lividans* TK24, in the strain deleted for *wblI* and in the strains carrying *wblI* overexpressing plasmid or the empty plasmid at 24, 48 and 72 h. All values were expressed as means ± SD (n = 5).

In order to assess *in vivo*, the impact of WblI on the expression of the target genes validated by EMSA, mRNA was prepared from the wild type strain of *S*. *lividans* TK24 carrying the empty vector or carrying the *wblI* over-expression plasmid or deleted for *wblI*, at 24, 48 and 72 h. The expression levels of *wblA*, *actVA3*, *actII-1* and *actII-2* were examined by qRT-PCR. Results are shown in Fig 4. In the wild type strain, the expression of genes of the ACT cluster (*actVA3*, *actII-1* and *actII-2*) was enhanced at 48 h, a time point where ACT was detectable. At the three time points tested, the over-expression of WblI was correlated with the increased expression of the genes encoding the hypothetical protein ActVA3 and the ACT transporter ActII-2, while transcription of the divergently located TetR regulator ActII-1 and of WblA was reduced. Consistently, the deletion of *wblI*, at all three time points, was correlated with reduced expression of *actVA3* and of *actII-2* and with enhanced expression of *actII-1* and *wblA*. Taken together with the above EMSA data, these results demonstrated that WblI positively regulates the transcription of *actVA3* while negatively controlling that of *actII-1* and *wblA via* direct interaction with their promoter regions.

### Overexpression of *wblI* enhances ACT export

Since the TetR regulator ActII-1 that regulates negatively ActII-2, the ACT export system, was proposed as a WblI regulatory target, the ratio between extracellular (exported) and intracellular ACT was determined in strains of *S*. *lividans* TK24 carrying the empty vector (pWHM3-ermE*) or the *wblI* overexpression plasmid (pWHM3- ermE*-wblI). This ratio was found to be 1.3 fold higher in the WblI overproducing strain than in the control strain indicating that WblI had a positive impact on ACT export (Table A in S1 Appendix).

## Discussion

The WhiB-like transcriptional regulator [35], WblI (SCO5046), studied in this issue was first identified as a target of the transcriptional regulator of the TetR-family (SCO3201). The over-expression of SCO3201 was shown to lead to strong repression of both antibiotic production and sporulation in *S*. *coelicolor* [31]. However, since the disruption of *SCO3201* did not lead to any obvious phenotype, it was proposed that SCO3201 “illegitimately” governs the expression of target genes normally under the control of other TetR regulators truly involved in the regulation of the differentiation process. It was thus inferred that the analysis of the “SCO3201” targets might lead to the identification of new players in the complex regulation of the differentiation process in *S*. *coelicolor*. Indeed, the search for SCO3021 targets allowed the identification of genes already known to be involved in the regulation of the differentiation process, validating this approach, as well as of new players including WblI (SCO5046).

In the present paper, we report the consequences of the over-expression of *wblI* on secondary metabolites production of *S*. *lividans* and the characterization of four of the putative regulatory targets of this regulator. The latter were identified using formaldehyde crosslinking followed by covalent capture of WblI/DNA complex on a Ni-NTA column, and were subsequently confirmed as direct targets by EMSA (Table 2, Fig 3) and qRT-PCR (Fig 4). Furthermore, an inspection of the promoter regions of the targets genes revealed some putative similar regulatory sequences. Altogether these investigations indicated that WblI represses its own transcription as well as that of the TetR regulator *actII-1* that represses the expression of the divergent gene *actII-2* encoding the ACT transporter [40]. qRT-PCR analysis confirmed that the transcription of *actII-1* was reduced whereas that of *actII-2* was enhanced in the WblI over-expressing strain. A putative WblI binding sequence was found to bracket the -10 region of *actII-1* suggesting repression of the expression of the latter by WblI. Consistently an enhanced ACT export was correlated with WblI over-expression. This enhanced export ought to be correlated with an enhanced ACT biosynthesis. The latter might be achieved *via* the positive effect that WblI exerts on the expression of *actVA3*, the third gene of a succession of six genes of the ActVA3 region (Fig 4). Indeed, three repeats of the putative WblI operator sequence were found upstream of the -35 promoter region of *act*VA3, in a position expected for an activator site. The function of ActVA3 is unknown but we propose that it might constitute a bottleneck in the ACT biosynthetic pathway. In the *wblI* over-expressing strain, its enhanced biosynthesis would thus contribute to observed enhanced ACT biosynthesis.

In contrast, the over-production of WblI was correlated with a reduction of the biosynthesis of the yellow mycelial pigment and of the grey spore pigment encoded by the CPK [42] and *whiE* loci, respectively (Fig 2). The synthesis of grey spore pigment, regarded as a sign of completion of spore maturation, is directed by the *whiE* locus composed of an operon of seven genes and a gene transcribed in the opposite direction [43,44]. The transcription of genes of this locus have been shown to rely on the presence on six known *whi* genes (including *whiB*), which are required for sporulation septum formation [44]. However, none of the *cpk* or *whiE* genes were identified as putative WblI target genes in our formaldehyde cross linking experiments (Table 2). This suggests that the negative impact that WblI has on these genes may be indirect. ACT, yCPK and WhiE are all polyketides so they are likely to compete for a common precursor, acetyl-CoA. Enhanced ACT biosynthesis would thus lead to decreased acetyl-CoA availability and thus reduced yCPK and grey pigment synthesis. This view is supported by the fact that the *wblI*-deletion mutant did not have any obvious phenotype in relation to yellow pigment production or spore pigmentation (Fig 2).

Finally and most importantly, our RT-PCR experiments revealed reduced expression of the *wblA* gene in the WblI over-expressing strain (Fig 4). In *S*. *coelicolor* the expression of WblA was shown to be under the negative control of AdpA [41], a regulator playing a positive role in aerial mycelium development and ACT production. The promoter region of WblA is very complex comprising 3 promoters and 6 putative AdpA binding motifs [41]. Interestingly, two direct repeats of a degenerated version of the putative WblI operator sequence CTTCGAS (gTTCGgG CC aTTCaAG) was noted between the P2 and P3 promoters of WblA (gTTCGgG CC aTTCaAG) in overlap of the binding site of AdpA (Fig 3B). This and our *in vivo* results suggested a negative regulation of WblA by WblI. WblA was shown to act as a negative regulator of ACT biosynthesis [24,41]. Consequently the repression by WblI, of WblA expression is consistent with enhanced ACT biosynthesis.

In *Mycobacteria tuberculosis*, Wbl proteins were shown to act as redox-sensing factors *via* their Fe-S clusters [45] and to trigger specific adaptive responses *via* their transcriptional regulator activity. The expression of the seven Wbl proteins (Whib 1–7) of this species was shown to be induced by various oxidative stresses [46]. Interestingly, in the WblA mutant of *S*. *coelicolor*, the oxidative stress response as well as RED and ACT biosynthesis were up-regulated [24–26]. This indicated that oxidative stress might be higher in this strain than in the original strain and suggested that oxidative stress might constitute an important signal to trigger antibiotic biosynthesis.

**Fig 5:**
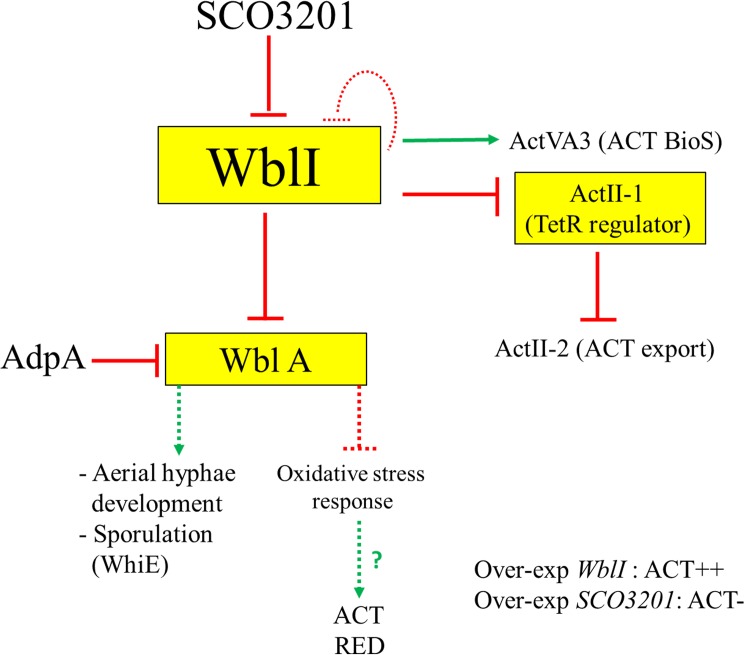
Schematic representation of the WblI and WblA regulatory network. Transcriptional regulators are boxed. Continuous and dotted lines represent proven direct or indirect regulatory interactions, respectively. Flat-headed and pointed arrows indicate repression and activation, respectively.

In summary, we provide in Fig 5 a schematic representation of the demonstrated WblI and WblA regulatory interactions. The strong enhancement that WblI over-expression has on ACT biosynthesis might result from the direct activation of ACT biosynthesis and export as well as to the repression of the expression of WblA, a likely indirect, repressor of ACT biosynthesis.

## Supporting information

S1 Appendix (includes Table A, Figure A and Figure B).
(DOCX)

## References

[1] Hopwood DA *Streptomyces* in Nature and Medicine The antibiotic makers Oxford university press, New York, NY 2007.

[2] Flardh K, Buttner MJ *Streptomyces* morphogenetics: dissecting differentiation in a filamentous bacterium. Nat Rev Microbiol.2009; 7(1): 36–49. 10.1038/nrmicro1968 19079351

[3] McCormick JR, Flardh K Signals and regulators that govern *Streptomyces* development. FEMS Microbiol Rev.2012; 36(1): 206–231. 10.1111/j.1574-6976.2011.00317.x 22092088 PMC3285474

[4] den Hengst CD, Tran NT, Bibb MJ, Chandra G, Leskiw BK, Buttner MJ Genes essential for morphological development and antibiotic production in *Streptomyces coelicolor* are targets of BldD during vegetative growth. Mol Microbiol.2010; 78(2): 361–379. 20979333 10.1111/j.1365-2958.2010.07338.x

[5] Higo A, Hara H, Horinouchi S, Ohnishi Y Genome-wide distribution of AdpA, a global regulator for secondary metabolism and morphological differentiation in *Streptomyces*, revealed the extent and complexity of the AdpA regulatory network. DNA Res.2012; 19(3): 259–273. 10.1093/dnares/dss010 22449632 PMC3372375

[6] Bibb MJ, Domonkos A, Chandra G, Buttner MJ Expression of the chaplin and rodlin hydrophobic sheath proteins in *Streptomyces venezuelae* is controlled by sigma(BldN) and a cognate anti-sigma factor, RsbN. Mol Microbiol.2012; 84(6): 1033–1049. 10.1111/j.1365-2958.2012.08070.x 22582857

[7] Chater KF, Chandra G The use of the rare UUA codon to define "expression space" for genes involved in secondary metabolism, development and environmental adaptation in *streptomyces*. J Microbiol.2008; 46(1): 1–11. 10.1007/s12275-007-0233-1 18337685

[8] Hackl S, Bechthold A The Gene *bldA*, a regulator of morphological differentiation and antibiotic production in *streptomyces*. Arch Pharm (Weinheim).2015; 348(7): 455–462. 25917027 10.1002/ardp.201500073

[9] Tschowri N, Schumacher MA, Schlimpert S, Chinnam NB, Findlay KC, Brennan RG, et al Tetrameric c-di-GMP mediates effective transcription factor dimerization to control *Streptomyces* development. Cell.2014; 158(5): 1136–1147. 10.1016/j.cell.2014.07.022 25171413 PMC4151990

[10] Bush MJ, Tschowri N, Schlimpert S, Flardh K, Buttner MJ c-di-GMP signalling and the regulation of developmental transitions in streptomycetes. Nat Rev Microbiol.2015; 13(12): 749–760. 10.1038/nrmicro3546 26499894

[11] Ryding NJ, Kelemen GH, Whatling CA, Flardh K, Buttner MJ, Chater KF A developmentally regulated gene encoding a repressor-like protein is essential for sporulation in *Streptomyces coelicolor* A3(2). Mol Microbiol.1998; 29(1): 343–357. 9701826 10.1046/j.1365-2958.1998.00939.x

[12] Ainsa JA, Parry HD, Chater KF A response regulator-like protein that functions at an intermediate stage of sporulation in *Streptomyces coelicolor* A3(2). Mol Microbiol.1999; 34(3): 607–619. 10564501 10.1046/j.1365-2958.1999.01630.x

[13] Persson J, Chater KF, Flardh K Molecular and cytological analysis of the expression of *Streptomyces* sporulation regulatory gene *whiH*. FEMS Microbiol Lett.2013; 341(2): 96–105. 10.1111/1574-6968.12099 23398592

[14] Kaiser BK, Stoddard BL DNA recognition and transcriptional regulation by the WhiA sporulation factor. Sci Rep.2011; 1: 156 10.1038/srep00156 22355671 PMC3240954

[15] Soliveri J, Brown KL, Buttner MJ, Chater KF Two promoters for the *whiB* sporulation gene of *Streptomyces coelicolor* A3(2) and their activities in relation to development. J Bacteriol.1992; 174(19): 6215–6220. 1400171 10.1128/jb.174.19.6215-6220.1992 PMC207690

[16] Soliveri JA, Gomez J, Bishai WR, Chater KF Multiple paralogous genes related to the Streptomyces coelicolor developmental regulatory gene whiB are present in Streptomyces and other actinomycetes. Microbiology.2000; 146 (Pt 2): 333–343.doi:10.1099/00221287-146-2-333. 10708372 10.1099/00221287-146-2-333

[17] Flardh K, Findlay KC, Chater KF Association of early sporulation genes with suggested developmental decision points in *Streptomyces coelicolor* A3(2). Microbiology.1999; 145 (Pt 9): 2229–2243.doi:10.1099/00221287-145-9-2229. 10517576 10.1099/00221287-145-9-2229

[18] Bush MJ, Chandra G, Bibb MJ, Findlay KC, Buttner MJ Genome-Wide Chromatin Immunoprecipitation Sequencing Analysis Shows that WhiB Is a Transcription Factor That Cocontrols Its Regulon with WhiA To Initiate Developmental Cell Division in *Streptomyces*. MBio.2016; 7(2).doi:10.1128/mBio.00523-16. 10.1128/mBio.00523-16 PMC4850268 27094333

[19] Smith LJ, Stapleton MR, Buxton RS, Green J Structure-function relationships of the *Mycobacterium tuberculosis* transcription factor WhiB1. PLoS One.2012; 7(7): e40407 10.1371/journal.pone.0040407 22792304 PMC3390391

[20] Zheng F, Long Q, Xie J The function and regulatory network of WhiB and WhiB-like protein from comparative genomics and systems biology perspectives. Cell Biochem Biophys.2012; 63(2): 103–108. 10.1007/s12013-012-9348-z 22388511

[21] Burian J, Yim G, Hsing M, Axerio-Cilies P, Cherkasov A, Spiegelman GB, et al The mycobacterial antibiotic resistance determinant WhiB7 acts as a transcriptional activator by binding the primary sigma factor SigA (RpoV). Nucleic Acids Res.2013; 41(22): 10062–10076. 10.1093/nar/gkt751 23990327 PMC3905903

[22] Casonato S, Cervantes Sanchez A, Haruki H, Rengifo Gonzalez M, Provvedi R, Dainese E, et al WhiB5, a transcriptional regulator that contributes to *Mycobacterium tuberculosis* virulence and reactivation. Infect Immun.2012; 80(9): 3132–3144. 10.1128/IAI.06328-11 22733573 PMC3418748

[23] Fowler-Goldsworthy K, Gust B, Mouz S, Chandra G, Findlay KC, Chater KF The actinobacteria-specific gene wblA controls major developmental transitions in Streptomyces coelicolor A3(2). Microbiology.2011; 157(Pt 5): 1312–1328. 10.1099/mic.0.047555-0 21330440

[24] Kang SH, Huang J, Lee HN, Hur YA, Cohen SN, Kim ES Interspecies DNA microarray analysis identifies WblA as a pleiotropic down-regulator of antibiotic biosynthesis in *Streptomyces*. J Bacteriol.2007; 189(11): 4315–4319. 10.1128/JB.01789-06 17416669 PMC1913423

[25] Kim JS, Lee HN, Kim P, Lee HS, Kim ES Negative role of *wblA* in response to oxidative stress in *Streptomyces coelicolor*. J Microbiol Biotechnol.2012; 22(6): 736–741. 22573149 10.4014/jmb.1112.12032

[26] Kim JS, Lee HN, Lee HS, Kim P, Kim ES A WblA-binding protein, SpiA, involved in *Streptomyces* oxidative stress response. J Microbiol Biotechnol.2013; 23(10): 1365–1371. 23867703 10.4014/jmb.1306.06032

[27] Molle V, Palframan WJ, Findlay KC, Buttner MJ WhiD and WhiB, homologous proteins required for different stages of sporulation in *Streptomyces coelicolor* A3(2). J Bacteriol.2000; 182(5): 1286–1295. 10671449 10.1128/jb.182.5.1286-1295.2000 PMC94414

[28] Crack JC, den Hengst CD, Jakimowicz P, Subramanian S, Johnson MK, Buttner MJ, et al Characterization of [4Fe-4S]-containing and cluster-free forms of *Streptomyces* WhiD. Biochemistry.2009; 48(51): 12252–12264. 10.1021/bi901498v 19954209 PMC2815329

[29] Jakimowicz P, Cheesman MR, Bishai WR, Chater KF, Thomson AJ, Buttner MJ Evidence that the *Streptomyces* developmental protein WhiD, a member of the WhiB family, binds a [4Fe-4S] cluster. J Biol Chem.2005; 280(9): 8309–8315. 10.1074/jbc.M412622200 15615709

[30] Homerova D, Sevcikova J, Kormanec J Characterization of the *Streptomyces coelicolor* A3(2) *wblE* gene, encoding a homologue of the sporulation transcription factor. Folia Microbiol (Praha).2003; 48(4): 489–495. 14533480 10.1007/BF02931330

[31] Xu D, Seghezzi N, Esnault C, Virolle MJ Repression of antibiotic production and sporulation in *Streptomyces coelicolor* by overexpression of a TetR family transcriptional regulator. Appl Environ Microbiol.2010; 76(23): 7741–7753. 10.1128/AEM.00819-10 20935121 PMC2988594

[32] Kieser T, Bibb M. J., Chater K., and Hopwood D. A. Practical Streptomyces genetics. The John Innes Foundation, Norwich, United Kingdom 2000.

[33] Sanchez J, Barbes C, Hernandez A, de los Reyes Gavilan CR, Hardisson C Restriction-modification systems in *Streptomyces antibioticus*. Can J Microbiol.1985; 31(10): 942–946. 2998580 10.1139/m85-177

[34] Bibb MJ, Janssen GR, Ward JM Cloning and analysis of the promoter region of the erythromycin resistance gene (*ermE*) of *Streptomyces erythraeus*. Gene.1985; 38(1–3): 215–226. 2998943 10.1016/0378-1119(85)90220-3

[35] Xu D, Waack P, Zhang Q, Werten S, Hinrichs W, Virolle MJ Structure and regulatory targets of SCO3201, a highly promiscuous TetR-like regulator of Streptomyces coelicolor M145. Biochem Biophys Res Commun.2014; 450(1): 513–518. 10.1016/j.bbrc.2014.06.003 24928397

[36] D'Alia D, Eggle D, Nieselt K, Hu WS, Breitling R, Takano E Deletion of the signalling molecule synthase ScbA has pleiotropic effects on secondary metabolite biosynthesis, morphological differentiation and primary metabolism in *Streptomyces coelicolor* A3(2). Microb Biotechnol.2011; 4(2): 239–251. 10.1111/j.1751-7915.2010.00232.x 21342469 PMC3818864

[37] Gottelt M, Kol S, Gomez-Escribano JP, Bibb M, Takano E Deletion of a regulatory gene within the *cpk* gene cluster reveals novel antibacterial activity in *Streptomyces coelicolor* A3(2). Microbiology.2010; 156(Pt 8): 2343–2353. 10.1099/mic.0.038281-0 20447997

[38] Pawlik K, Kotowska M, Kolesinski P *Streptomyces coelicolor* A3(2) produces a new yellow pigment associated with the polyketide synthase Cpk. J Mol Microbiol Biotechnol.2010; 19(3): 147–151. 10.1159/000321501 20924201

[39] Caballero JL, Martinez E, Malpartida F, Hopwood DA Organisation and functions of the *actVA* region of the actinorhodin biosynthetic gene cluster of *Streptomyces coelicolor*. Mol Gen Genet.1991; 230(3): 401–412. 1766437 10.1007/BF00280297

[40] Caballero JL, Malpartida F, Hopwood DA Transcriptional organization and regulation of an antibiotic export complex in the producing *Streptomyces* culture. Mol Gen Genet.1991; 228(3): 372–380. 1716725 10.1007/BF00260629

[41] Lee HN, Kim JS, Kim P, Lee HS, Kim ES Repression of antibiotic downregulator WblA by AdpA in *Streptomyces coelicolor*. Appl Environ Microbiol.2013; 79(13): 4159–4163. 10.1128/AEM.00546-13 23603676 PMC3697564

[42] Pawlik K, Kotowska M, Chater KF, Kuczek K, Takano E A cryptic type I polyketide synthase (*cpk*) gene cluster in *Streptomyces coelicolor* A3(2). Arch Microbiol.2007; 187(2): 87–99. 10.1007/s00203-006-0176-7 17009021

[43] Davis NK, Chater KF Spore colour in *Streptomyces coelicolor* A3(2) involves the developmentally regulated synthesis of a compound biosynthetically related to polyketide antibiotics. Mol Microbiol.1990; 4(10): 1679–1691. 2077356 10.1111/j.1365-2958.1990.tb00545.x

[44] Kelemen GH, Brian P, Flardh K, Chamberlin L, Chater KF, Buttner MJ Developmental regulation of transcription of *whiE*, a locus specifying the polyketide spore pigment in *Streptomyces coelicolor* A3 (2). J Bacteriol.1998; 180(9): 2515–2521. 9573206 10.1128/jb.180.9.2515-2521.1998 PMC107196

[45] Singh A, Guidry L, Narasimhulu KV, Mai D, Trombley J, Redding KE, et al *Mycobacterium tuberculosis* WhiB3 responds to O2 and nitric oxide via its [4Fe-4S] cluster and is essential for nutrient starvation survival. Proc Natl Acad Sci U S A.2007; 104(28): 11562–11567. 10.1073/pnas.0700490104 17609386 PMC1906726

[46] Larsson C, Luna B, Ammerman NC, Maiga M, Agarwal N, Bishai WR Gene expression of *Mycobacterium tuberculosis* putative transcription factors *whiB1-7* in redox environments. PLoS One.2012; 7(7): e37516 10.1371/journal.pone.0037516 22829866 PMC3400605

