# Supplementary material for: In conditions of over-expression, WblI, a WhiB-like transcriptional regulator, has a positive impact on the weak antibiotic production of Streptomyces lividans TK24
Source: PLoS One. 2017 Mar 30;12(3):e0174781. doi: 10.1371/journal.pone.0174781 (PMC5373594; doi:10.1371/journal.pone.0174781)
Supplement: S1 Appendix — (includes Table A, Figure A and Figure B). (DOCX) [file pone.0174781.s001.docx]

**PLoS One**

**In conditions of over-expression, WblI, a WhiB-like transcriptional regulator, has a positive impact on the weak antibiotic production of *Streptomyces lividans* TK24.**

Lan Yan ^1^, Qizhong Zhang ^1^, Marie-Joelle Virolle ^2^ **^†^*** and Delin Xu ^1^**^†^***

* Corresponding authors

**^†^** These corresponding authors contributed equally to this work.

**^1^** Department of Ecology, Institute of Hydrobiology, School of Life Science and Technology, Key Laboratory of Eutrophication and Red Tide Prevention of Guangdong Higher Education Institutes, Engineering Research Center of Tropical and Subtropical Aquatic Ecological Engineering, Ministry of Education, Jinan University, Guangzhou, 510632, PR China

**^2^** Group "Energetic Metabolism of *Streptomyces* ", Institute for Integrative Biology of the Cell (I2BC), CEA, CNRS, Univ**.** Paris‐Sud, INRA, Université Paris‐Saclay, F-91198, Gif‐sur‐Yvette Cedex, France

**Email addresses:** [xudelin@hotmail.com](mailto:xudelin@hotmail.com) (D. Xu),

[marie-joelle.virolle@i2bc.paris-saclay.fr](mailto:marie-joelle.virolle@i2bc.paris-saclay.fr%20)  (M.-J. Virolle)

**Tel:** (0086)-20-85225808 (D. Xu), (0033)-169156913 (M.-J. Virolle)

**Fax:** (0033)-169154642 (M.-J. Virolle)

**Table A. Quantitative analysis of actinorhodin (intracellular and extracellular values)**

| **Concn (µmol/g [dry wt] cells)^α^** | | | | | | |
| --- | --- | --- | --- | --- | --- | --- |
| ***S. lividans* TK24/pWHM3** | | |  | ***S. lividans* TK24/pWHM3-*wblI*** | | |
| **In** | **Out** | **Ratio (out/in)** |  | **In** | **Out** | **Ratio (out/in)** |
| 8.4 ± 0.23 | 12.6 ± 0.51 | 1.5 |  | 165.25 ± 7.8 | 322.34 ± 1.61 | 1.95 |

^α^ In, intracellular, Out, extracellular.

**Figure A.
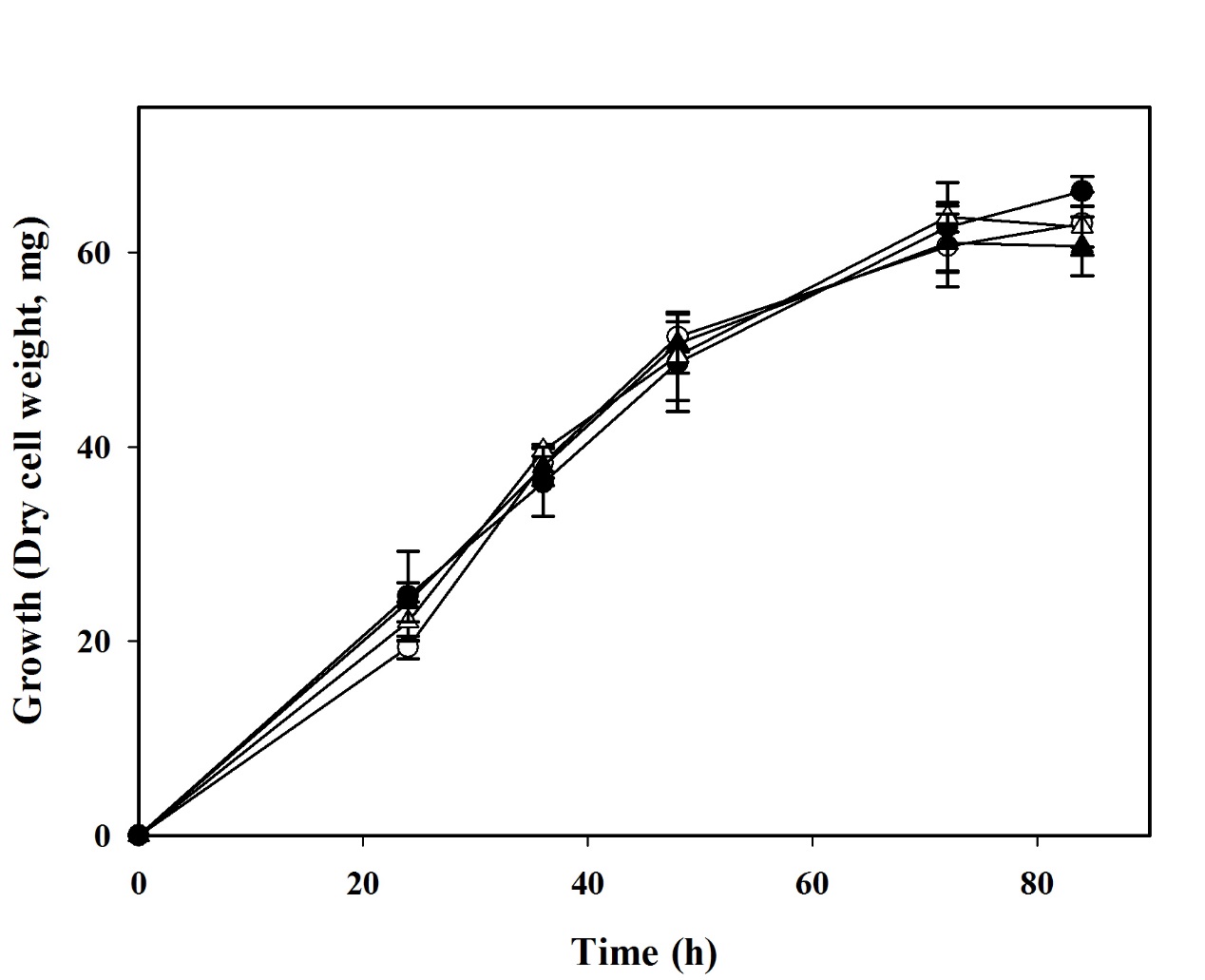
**

**Figure A.** Growth curves of the wild type strain of *S. lividans* TK24 (●), of the strain deleted for *wblI* (○), of the strains carrying *wblI* overexpressing plasmid (▲) and empty vector (△).

**Figure B.**

**
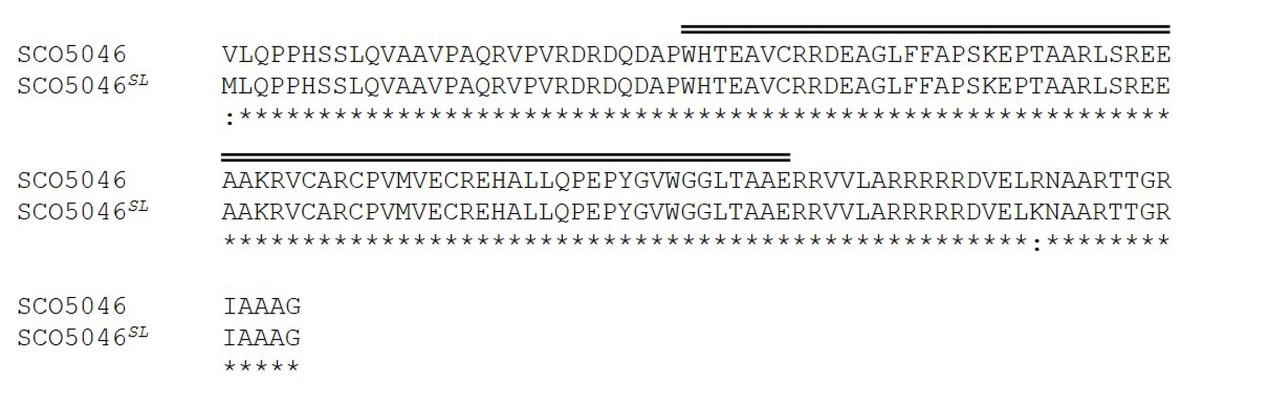
**

**Figure B.** Alignment of the SCO5046 amino acid sequences from *S. coelicolor* M145 and *S. lividans* TK24. The conserved region of the WhiB family proteins are marked by over double-lines.
